# DISCRIMINATION AND COPING AMONG OLDER CHINESE IMMIGRANTS DURING THE COVID-19 PANDEMIC: A QUALITATIVE STUDY

**DOI:** 10.1093/geroni/igac059.250

**Published:** 2022-12-20

**Authors:** Jiaming Liang, Kexin Yu, Yi-Hsuan Tung, Choi Ha Li, Shinyi Wu, Iris Chi

**Affiliations:** University of Southern California, Los Angeles, California, United States; Oregon Health & Science University, Palo Alto, California, United States; University of Southern California, Los Angeles, California, United States; University of Southern California, Los Angeles, California, United States; University of Southern California, Los Angeles, California, United States; University of Southern California, Los Angeles, California, United States

## Abstract

Discriminatory events against Asians, especially Chinese, became rampant during the COVID-19 pandemic. It is difficult for older Chinese immigrants to effectively protect themselves from racism-related attacks due to their personal and social disadvantages. This study explored older Chinese immigrants’ experience of discrimination and coping strategies, as well as factors that influence their perceptions, attitudes, and coping preferences. Among 27 interviewees, 11 experienced discriminatory incidents themselves or known people around had been discriminated against during the pandemic. Thematic analysis revealed negative psychological impact of discrimination risk or experience. Most participants tended to adopt disengagement coping styles, such as avoidance, rationalization, and reducing social participation. Three primary influencing factors are: (1) perceived unkindness from government and public opinions; (2) concern for own health; (3) limited acculturation. Our findings suggest needing efforts to protect the safety of older Chinese immigrants, and raise their awareness and ability to defend themselves from racism and discrimination.

